# Heterologous Rhamnolipid Biosynthesis: Advantages, Challenges, and the Opportunity to Produce Tailor-Made Rhamnolipids

**DOI:** 10.3389/fbioe.2020.594010

**Published:** 2020-10-22

**Authors:** Andreas Wittgens, Frank Rosenau

**Affiliations:** ^1^Institute of Pharmaceutical Biotechnology, Ulm University, Ulm, Germany; ^2^Ulm Center for Peptide Pharmaceuticals (U-PEP), Ulm University, Ulm, Germany; ^3^Department Synthesis of Macromolecules, Max-Planck-Institute for Polymer Research Mainz, Mainz, Germany

**Keywords:** rhamnolipids, biosurfactants, *Pseudomonas putida*, *Pseudomonas aeruginosa*, *Burkholderia glumae*, heterologous production, *quorum sensing*

## Abstract

The first heterologous expression of genes responsible for the production of rhamnolipids was already implemented in the mid-1990s during the functional identification of the *rhlAB* operon. This was the starting shot for multiple approaches to establish the rhamnolipid biosynthesis in different host organisms. Since most of the native rhamnolipid producing organisms are human or plant pathogens, the intention for these ventures was the establishment of non-pathogenic organisms as heterologous host for the production of rhamnolipids. The pathogenicity of producing organisms is one of the bottlenecks for applications of rhamnolipids in many industrial products especially foods and cosmetics. The further advantage of heterologous rhamnolipid production is the circumvention of the complex regulatory network, which regulates the rhamnolipid biosynthesis in wild type production strains. Furthermore, a suitable host with an optimal genetic background to provide sufficient amounts of educts allows the production of tailor-made rhamnolipids each with its specific physico-chemical properties depending on the contained numbers of rhamnose sugar residues and the numbers, chain length and saturation degree of 3-hydroxyfatty acids. The heterologous expression of *rhl* genes can also enable the utilization of unusual carbon sources for the production of rhamnolipids depending on the host organism.

## Introduction

Rhamnolipids are among the best characterized biosurfactants and possess outstanding properties to replace or complement conventional surfactants based on petrochemistry in industrial and biotechnological applications. Rhamnolipids are considered as eco-friendly and sustainable, show a very low toxicity, are highly or even perfectly biocompatible and biodegradable (Desai and Banat, 1997; van Hamme et al., 2006; Hirata et al., 2009; Lima et al., 2011; Johann et al., 2016). As an important additional aspect, rhamnolipids exclusively originate from microbiological hosts and can be produced from renewable substrates in contrast to their synthetical counterparts, which primary base on fossil resources (Müller et al., 2010b). Combined with their better foaming properties and remarkable stabilities against extreme pH-values, temperatures and salt concentrations, rhamnolipids offer a broad application potential (Lang and Wullbrandt, 1999; Nitschke and Pastore, 2006; Banat et al., 2010). Rhamnolipids are used in traditional surfactant applications like washing detergents and cleaning agents (Nguyen and Sabatini, 2011), but also in cosmetics and foods (Maier and Soberón-Chávez, 2000; Nitschke and Costa, 2007), bioremediation (Nguyen et al., 2008; Liu et al., 2018) and (microbial) enhanced oil recovery (Wang et al., 2007; Sharma et al., 2018).

**FIGURE 1:**
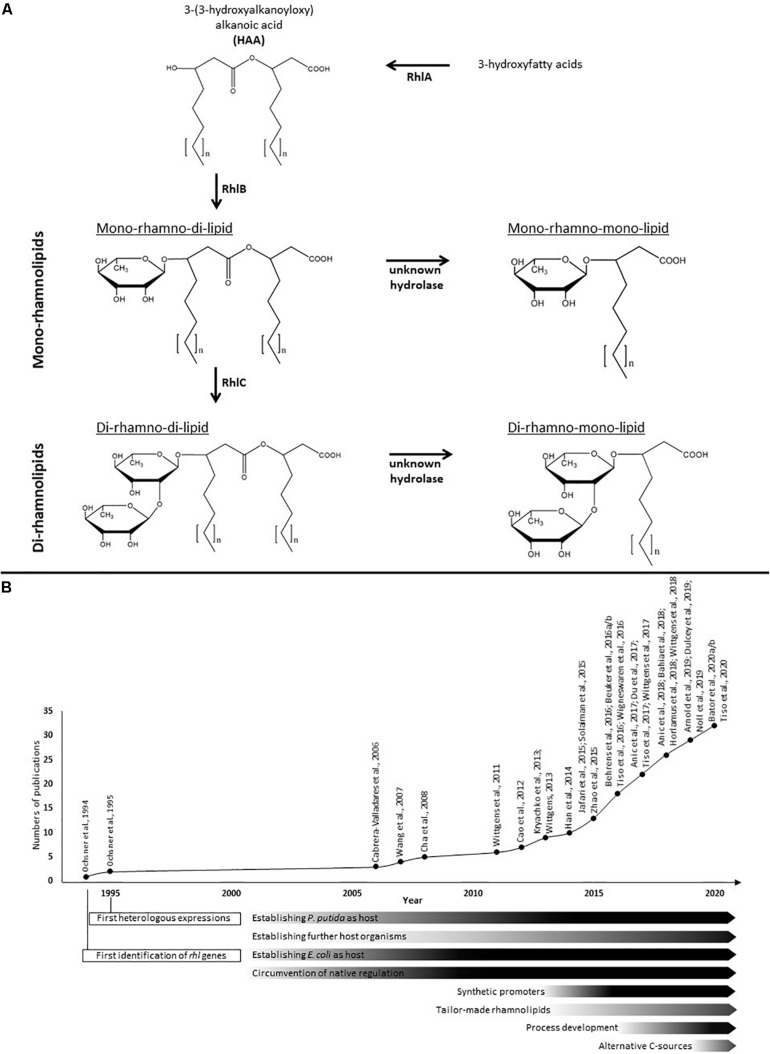
Rhamnolipid biosynthesis and research efforts in heterologous rhamnolipid production. **(A)** The biosynthesis of rhamnolipids occurs in consecutive enzymatic reactions. The esterification of two 3-hydroxyfatty acids is catalyzed by the acyltransferase RhlA and generates HAAs as rhamnolipid precursors. The rhamnosyltransferase I (RhlB) generates mono-rhamnolipids by adding a dTDP-L-rhamnose to the HAAs. Di-rhamnolipids are synthesized by the rhamnosyltransferase II (RhlC) by linking a second dTDP-L-rhamnose to the mono-rhamnolipids. Both rhamnolipid species can be further processed by hydrolases to create mono-rhamno-mono-lipids and di-rhamno-mono-lipids containing only one fatty acid chain. The fatty acids chain lengths of rhamnolipids typically vary between C_8_ and C_16_. **(B)** Time course of efforts and milestones in the research of heterologous rhamnolipid production.

The amphiphilic rhamnolipids belong to the class of glycolipids and are composed of a hydrophilic molecule domain consisting of one or two L-rhamnoses β-glycosidically bound to a hydrophobic counterpart consisting of one or two 3-hydroxyfatty acids (Figure 1; Déziel et al., 1999; Abdel-Mawgoud et al., 2010). Based on the number of rhamnose residues, they are separated into mono- and di-rhamnolipids. The number of 3-hydroxyfatty acids they contain allows a further sub-classification into the four known species of natively occurring rhamnolipids, namely the predominant mono- and di-rhamno-**di**-lipids (mRdL and dRdL) and the rarer to find mono-and di-rhamno-**mono**-lipids (mRmL and dRmL) (Syldatk et al., 1985a,b; Déziel et al., 1999; Abdel-Mawgoud et al., 2010). The chain length of the fatty acids can vary within the species ranging typically from 8 to 16 carbon atoms depending on the producing wild type. Short-chain rhamnolipids containing a predominant C_10_-C_10_ hydrophobic moiety are produced in the highest concentrations known so far by the human-pathogenic organism *Pseudomonas aeruginosa* (Giani et al., 1997; Müller et al., 2010a) and were first described by Jarvis and Johnson (1949). Among a few other organisms, especially bacteria from the genus *Burkholderia*, are able to produce rhamnolipids with long-chain fatty acids and predominant C_14_-C_14_ congener (Häußler et al., 1998; Andrä et al., 2006; Funston et al., 2016). In organisms naturally producing rhamnolipids are essential for swarming motility, involved in biofilm formation and act as hemolysins (Köhler et al., 2000; Davey et al., 2003; Tremblay et al., 2007). In addition, they enhance the uptake of hydrophobic substrates (Zhang and Miller, 1995; Al-Tahhan et al., 2000; Noordman and Janssen, 2002) and play a role in shielding the producing cells from host defense (McClure and Schiller, 1996; van Gennip et al., 2009; Alhede et al., 2009).

The biosynthesis of rhamnolipids is initiated by the esterification of two 3-hydroxyfatty acids to form the rhamnolipid precursor molecules 3-(3-hydroxyalkanoyloxy)alcanoic acids (HAAs), which typically form the hydrophobic moiety of the final rhamnolipids. This dimerization is catalyzed by the acyltransferase RhlA (Déziel et al., 2003; Zhu and Rock, 2008). It is controversially discussed, whether the 3-hydroxyfatty acids are descending from the fatty acid *de novo* synthesis while bound to the acyl carrier protein (Rehm et al., 2001), as it has been described earlier to be the exclusively accepted substrate of RhlA (Zhu and Rock, 2008), or if the β-oxidation is the main provider of 3-hydroxyfatty acids for the HAA and rhamnolipid biosynthesis (Zhang et al., 2012; Abdel-Mawgoud et al., 2014). An HAA molecule together with one molecule of dTDP-L-rhamnose descending from glucose-6-phosphate (Olvera et al., 1999; Rahim et al., 2000) are used as substrates for the biosynthesis of mono-rhamnolipids by the rhamnosyltransferase I (RhlB) (Ochsner et al., 1994; Wittgens et al., 2017). Finally, di-rhamnolipids are synthesized by adding a second dTDP-L-rhamnose molecule to the mono-rhamnolipids by the rhamnosyltransferase II (RhlC) (Rahim et al., 2001). Subsequently, the biosynthesis of mono-rhamno-mono-lipids and di-rhamno-mono-lipids can occur through hydrolysis of the second 3-hydroxyacyl chain probably by two specific but yet unknown α/β-hydrolases (Wittgens et al., 2017). In *P. aeruginosa* the *rhlA* and *rhlB* genes are organized within an operon (Ochsner et al., 1994), while *rhlC* is part of a second operon encoded together with *PA1131*, which shares similarities with transport proteins of the major facilitator superfamily (MFS) (Rahim et al., 2001). However, PA1131 appeared to be not involved in rhamnolipid biosynthesis or secretion (Wittgens et al., 2017). In contrast, in *Burkholderia* species all *rhl* genes are located within a single gene cluster (Dubeau et al., 2009; Lim et al., 2009).

Apart from the natural rhamnolipid producer organisms an increasing number of different recombinant hosts was generated by introduction of *rhl* genes. In this review, we will give an update of different approaches for the heterologous production of diverse rhamnolipids from the functional identification of the *rhlAB* operon in the 1990s to efficient modern production strains. We explain the reasons for this concept as well as the challenges to find a suitable host organism and the current strategies to synthesize tailor-made rhamnolipids. Thereby, we focus on real heterologous expression of foreign genes in recombinant hosts rather than metabolic engineering of wild type rhamnolipid producers.

## Heterologous Production of Rhamnolipids—Advantages and Challenges

The overall arguments for a heterologous expression of target genes—in this case the *rhl* genes responsible for the biosynthesis of rhamnolipids—are numerous. The first implementation was performed to finally proof the responsibility of the *rhlAB* operon for the mono-rhamnolipid biosynthesis. After the successful identification of the *rhlAB* operon in studies with *P. aeruginosa* mutant strains Ochsner et al. (1994) expressed single *rhl* genes and operons heterologously in *Escherichia coli* DH5α (Table 1). While the evidence for a rhamnosyltransferase activity using 3-hydroxydecanoyl-3-hydroxydecanoate and TDP-rhamnose as substrate was positive, they could not detect any production of rhamnolipids in *E. coli*.

**TABLE 1:** Summary of approaches for the heterologous production of rhamnolipids.

**Host organism**	**Heterologous genes/operons**	**Controlling promotor**	**Source/donor organism**	**Max. titers (g/L)**	**References**
*Burkholderia glumae* BGR1 (Δ*rhlA*)	*rhlA*_*Pa*_-*rhlB*_*Pa*_	*lac*-promoter, constitutive	*Pseudomonas aeruginosa* PA14/*Burkholderia glumae* BGR1	n. d.	Dulcey et al., 2019
	*rhlA*_*Bg*_-*rhlB*_*Pa*_				
	*rhlA*_*Pa*__/_*_*Bg*_*-*rhlB*_*Pa*_				
*Cellvibrio japonicus* Ueda107	*rhlAB*	Synthetic promoter, constitutive	*Pseudomonas aeruginosa* PAO1	4.90	Horlamus et al., 2018
*Escherichia coli* BL21(DE3)	*rhlA*, *rhlB*, *rhlC*, *rhlAB*, *rhlAC*, *rhlBC*, *rhlABC*, *rhlA*-*rhlB* L168X	T7-promoter, IPTG inducible	*Pseudomonas aeruginosa* PAO1	0.12	Han et al., 2014
	*rhlAB*	T7-promoter, IPTG inducible	*Pseudomonas aeruginosa* PAO1	0.18	Wang et al., 2007
	*rhlAB*	T7-promoter, IPTG inducible	*Pseudomonas aeruginosa* ATCC 9027	n. d.	Jafari et al., 2014
	*rhlAB*_*Pa*_-*rhlC*_*Pa*_	T7-promoter, IPTG inducible	*Pseudomonas aeruginosa* PAO1*/Burkholderia pseudomalii* K96243	0.64	Du et al., 2017
	*rhlAB*_*Bp*_-*rhlC*_*Bp*_				
	*rhlAB*_*Pa*_-*rhlC*_*Bp*_				
	*rhlAB*_*Bp*_-*rhlC*_*Pa*_				
	*rhlAB*	T5-/*lac*-/*ara*-/Trc-promoter, IPTG inducible	*Pseudomonas aeruginosa* PAO1	n. d.	Du et al., 2017
	*rhlC*	T7-promoter, IPTG inducible			
*Escherichia coli* DH5α	*rhlA*, *rhlB*,	*tac*-promoter, IPTG inducible	*Pseudomonas aeruginosa* PG201	<0.001	Ochsner et al., 1994
	*rhlAB*, *rhlAB*-*rhlR*				
	*rhlAB-rhlR-rhlI*	*rhl*-promoter, native RhlR/I regulation	*Pseudomonas aeruginosa* PG201	<0.02	Ochsner et al., 1995
	*rhlAB*	*tac*-promoter, IPTG inducible	*Pseudomonas aeruginosa* PG201	<0.02	Ochsner et al., 1995
*Escherichia coli* HB101	*rhlAB-rhlR*	*tac*-promoter, IPTG inducible	*Pseudomonas aeruginosa* PG201	0.05	Cabrera-Valladares et al., 2006
*Escherichia coli* TG2	*rhlAB*	*lac*-promoter, constitutive	Pseudomonas aeruginosa PA14	n. d.	Kryachko et al., 2013
*Escherichia coli* W3110	*rhlAB-rhlR*	*tac*-promoter, IPTG inducible	*Pseudomonas aeruginosa* PG201	0.12	Cabrera-Valladares et al., 2006
*Escherichia coli* XL1-blue	*rhlA*, *rhlAB*	*tac*-promoter, IPTG inducible	*Pseudomonas aeruginosa* PG201	<0.001	Ochsner et al., 1994
*Pseudomonas aeruginosa* PA14 (Δ*rhlA*)	*rhlA*_*Bg*_-*rhlB*_*Pa*_	*lac*-promoter, constitutive	*Pseudomonas aeruginosa* PA14/*Burkholderia glumae* BGR1	n. d.	Dulcey et al., 2019
	*rhlA*_*Pa*__/_*_*Bg*_*-*rhlB*_*Pa*_				
*Pseudomonas chlororaphis* NRRL B-30761	*rhlC*	*Pseudomonas syringae* promoter, constitutive	*Pseudomonas aeruginosa* PAO1	0.24	Solaiman et al., 2015
*Pseudomonas fluorescens* ATCC 15453	*rhlAB-rhlR-rhlI*	*rhl*-promoter, native RhlR/I regulation	*Pseudomonas aeruginosa* PG201	0.25	Ochsner et al., 1995
	*rhlAB*	*tac*-promoter, IPTG inducible	*Pseudomonas aeruginosa* PG201	<0.02	Ochsner et al., 1995
*Pseudomonas oleovorans* GPo1	*rhlAB-rhlR-rhlI*	*rhl*-promoter, native RhlR/I regulation	*Pseudomonas aeruginosa* PG201	<0.02	Ochsner et al., 1995
	*rhlAB*	*tac*-promoter, IPTG inducible	*Pseudomonas aeruginosa* PG201	<0.02	Ochsner et al., 1995
*Pseudomonas putida* KCTC 1067	*rhlAB-rhlR-rhlI*	*rhl*-promoter, native RhlR/I regulation	*Pseudomonas aeruginosa* EMS1	7.30	Cha et al., 2008
*Pseudomonas putida* KT2442	*rhlAB-rhlR-rhlI*	*rhl*-promoter, native RhlR/I regulation	*Pseudomonas aeruginosa* PG201	<0.02	Ochsner et al., 1995
	*rhlAB*	*tac*-promoter, IPTG inducible	*Pseudomonas aeruginosa* PG201	0.60	Ochsner et al., 1995
*Pseudomonas putida* KT2440	*rhlAB*	*tac*-promoter, IPTG inducible	*Pseudomonas aeruginosa* PAO1	0.22 2.20	[Bibr B116][Bibr B100]
	*rhlAB-rhlR-rhlI*	*rhl*-promoter, native RhlR/I regulation	*Pseudomonas aeruginosa* BSFD5	1.68	Cao et al., 2012
	*rhlAB*	Synthetic promoter (library), constitutive	*Pseudomonas aeruginosa* PAO1	0.88	Wittgens, 2013
				n. d.	Behrens et al., 2016
				14.90	Beuker et al., 2016a,b
				3.20	Tiso et al., 2016, 2020
				≈0.01	Wigneswaran et al., 2016
				6.00	Anic et al., 2017, 2018
				0.83	Arnold et al., 2019
				1.20	Noll et al., 2019
				0.90	Bator et al., 2020a,b
	*rhlABC*	*tac*-promoter, IPTG inducible	*Pseudomonas aeruginosa* PAO1	n. d.	Behrens et al., 2016
	*rhlAB, rhlABC*	Synthetic promoter library, constitutive	*Pseudomonas aeruginosa* PAO1	3.30	Tiso et al., 2017
	*rhlA, rhlB, rhlC, rhlAB, rhlABC, rhlA* S102A*-rhlB*	*tac*-promoter, IPTG inducible	*Pseudomonas aeruginosa* PAO1	0.01	Wittgens et al., 2017
	*rhlAB_*Bg*_, rhlABC_*Bg*_*, *rhlC*_*Bg*_ *rhlA_*Pa*_rhlB_*Bg*_, rhlA_*Bg*_rhlB_*Pa*_*	*tac*-promoter, IPTG inducible	*Burkholderia glumae* PG1/*Pseudomonas aeruginosa* PAO1	0.08	Wittgens et al., 2018
	*rhlAB*	nagAa-promoter, salicylate inducible	*Pseudomonas aeruginosa* PAO1	1.30	Tiso et al., 2020
*Pseudomonas putida* KT2440 (Δflag), (Δ*phaG*), (Δpha), and (Δ*phaG*Δpha)	*rhlAB*	Synthetic promoter, constitutive	*Pseudomonas aeruginosa* PAO1	1.50	Tiso et al., 2020
*Pseudomonas putida* KT40CZC (Δ*pha*)	*rhlAB*	*tac*-promoter, IPTG inducible	*Pseudomonas aeruginosa* PAO1	2.40	Tiso et al., 2016
*Pseudomonas putida* KT42C1 (Δ*phaC1*)	*rhlAB*	*tac*-promoter, IPTG inducible	*Pseudomonas aeruginosa* PAO1	1.50	Wittgens et al., 2011
*Pseudomonas taiwanensis* VLB120	*rhlAB*	Synthetic promoter, constitutive	*Pseudomonas aeruginosa* PAO1	≈0.74	Tiso et al., 2017
*Pseudomonas stutzeri* DQ1	*rhlAB-rhlR-rhlI*	*rhl*-promoter, native RhlR/I regulation	*Pseudomonas aeruginosa* SQ6	1.61	Zhao et al., 2015
*Saccharomyces cerevisiae*	*rhlA*	TEF-promoter	*Pseudomonas aeruginosa*	n. d.	Bahia et al., 2018
CEN-PK 102-3A/CEN-PK 113-6B	*rhlB*	ADH-promoter			

*n. d., no data.*

A further reason for heterologous expression is to overcome the pathogenicity of most of the native producing organisms, which is disadvantageous for many industrial applications especially in foods and cosmetics (Toribio et al., 2010; Müller and Hausmann, 2011). The best known rhamnolipid producer *P. aeruginosa* as well as some rhamnolipid producing species of the genus *Burkholderia* like *B. pseudomallei* (Häußler et al., 1998, 2003) are human-pathogens, others like *B. plantarii* and *B. glumae* are at least plant-pathogens (Manso Pajarron et al., 1993; Andrä et al., 2006; Hörmann et al., 2010; Costa et al., 2011). Therefore, already in 1995 Ochsner et al. established the heterologous expression of *rhlAB* in four different organisms (Table 1). The operon was either controlled by an inducible *tac*-promoter or transcriptionally regulated by RhlR/I being part of a recombinant *rhlABRI* gene cluster. However, a reliable induction of *rhlAB* especially through the native regulation could not be achieved in all of the hosts, e.g., in *E. coli*. Nevertheless, the successful production of considerable amounts of mono-rhamnolipids after expression of *rhlABRI* was demonstrated for *Pseudomonas putida* (Cha et al., 2008; Cao et al., 2012) and *Pseudomonas stutzeri* (Zhao et al., 2015) as well (Table 1). This strategy certainly avoided only a part of the complex regulation system for the rhamnolipid biosynthesis. In *P. aeruginosa* the cell density depending *quorum sensing* system consisting primarily of LasR/I and RhlR/I as its central components is involved in the transcriptional regulation of both *rhl* operons (Williams and Cámara, 2009; Reis et al., 2011). Although they seem to be hierarchical organized, both systems are able to induce the expression of several genes including the *rhl* genes independent from each other (Latifi et al., 1996; Schuster and Greenberg, 2006). Experiments using *P. aeruginosa* mutant strains revealed a reduction of rhamnolipids of about 55%, when RhlI was absent, but even of almost 80% in the absence of LasI (Pearson et al., 1997), because its synthesized autoinducer bound to LasR is one of at least four regulators, which can induce the *rhlR* expression (Medina et al., 2003). In contrast, in LasR deficient mutants, the rhamnolipid biosynthesis is delayed, but finally reached concentrations like the wild type (Dekimpe and Déziel, 2009). Aside, further systems are existing being responsible to modulate the *quorum sensing* response and subsequent the rhamnolipid biosynthesis, e.g., the PQS system as the so-called third *quorum sensing* system (Pesci et al., 1999), the *quorum quenching* (Sio et al., 2006), the global regulators RsaL and Vfr (Rampioni et al., 2007, 2009; Croda-García et al., 2011) and further signaling systems (Wilhelm et al., 2007; Rosenau et al., 2010; Henkel et al., 2013), which is often depending on the cultivation conditions (Duan and Surette, 2007). Similar complex regulatory systems were also described for rhamnolipid producing *Burkholderia* (Nickzad et al., 2015; Nickzad and Déziel, 2016).

Since their regulation is one of the bottlenecks for the high-yield production of rhamnolipids (Toribio et al., 2010; Müller and Hausmann, 2011), it seems to be very challenging to enhance rhamnolipid production also in specific native producers, which are characterized as non-pathogenic like the *P. aeruginosa* strain ATCC 9027 (Grosso-Becerra et al., 2016) or species like *P. chlororaphis* (Gunther et al., 2005, 2006), *B. thailandensis* (Dubeau et al., 2009; Funston et al., 2016; Elshikh et al., 2017) and *B. kururiensis* (Tavares et al., 2012). Noteworthy, the non-pathogenic *P. chlororaphis* produce only mono-rhamnolipids and was complemented for di-rhamnolipid biosynthesis by heterologous expression of *rhlC* (Solaiman et al., 2015). However, almost none of these components exist in *E. coli* and, therefore, cannot positively affect the expression of *rhlR*/I or *rhlAB*. On the other hand, this gene cluster is not repressed by any influence and its successful sole native expression was repeatedly demonstrated (Table 1).

More recent studies focused on complete decoupling of heterologous *rhlAB* and *rhlC* expression from the native regulation. Especially for applications in the (microbial) enhanced oil recovery, rhamnolipids were produced in *E. coli* using the common T7 expression system (Wang et al., 2007; Han et al., 2014; Jafari et al., 2014; Du et al., 2017), while others established a constitutive expression of *rhlAB* in *E. coli* (Kryachko et al., 2013). More frequently, *P. putida* KT2440 wild type or engineered strains were used as heterologous host for rhamnolipid biosynthesis using an inducible *tac*-promoter or constitutive expressed and partly synthetic promoters (Wittgens et al., 2011, 2017; Wittgens, 2013; Behrens et al., 2016; Beuker et al., 2016a,b; Tiso et al., 2016, 2017, 2020; Anic et al., 2017, 2018; Noll et al., 2019; Table 1). Besides well-known short-chain rhamnolipids from *P. aeruginosa* also the heterologous production of long chain rhamnolipids was established in this organism by expressing *rhlAB* and *rhlC* from *B. glumae* (Wittgens et al., 2018).

Except typical strategies to enable heterologous expression in a specific host, e.g., codon usage optimization, application of compatible shuttle vectors and promoters, the availability of educts for the rhamnolipid biosynthesis is a challenging bottleneck. Thereby, especially the amount of dTDP-L-rhamnose seems to limit the productivity of rhamnolipids as it was shown in recombinant *E. coli* strains. Coexpression of the *rmlBDAC* operon, which converts glucose-1-phosphate into dTDP-L-rhamnose solved this problem and increased the rhamnolipid titers (Cabrera-Valladares et al., 2006).

Other researchers left the domain of bacteria and expressed codon-optimized genes responsible for the biosynthesis of mono-rhamnolipids in *Saccharomyces cerevisiae* (Bahia et al., 2018). As described for *E. coli*, they also had to use coexpression of the *rmlBDAC* operon to enable sufficient amounts of dTDP-L-rhamnose.

Recent studies are more focused on establishing unusual carbon sources or cultivation conditions to make the rhamnolipid biosynthesis more economical, e.g., by using cheap raw materials from waste streams. These studies were based on well-established host organisms like *P. putida* KT2440 engineered to utilize ethanol, pyrolysis oil or alternative sugars like xylose and arabinose as part of lignocellulosic hydrolysates or from agricultural residues (Arnold et al., 2019; Horlamus et al., 2019; Wang et al., 2019; Bator et al., 2020a,b) or they were cultivated in biofilms to avoid foaming as in conventional bioreactors (Wigneswaran et al., 2016). Moreover, new heterologous hosts for rhamolipid production were exploited, e.g., *Cellvibrio japonicus* (Horlamus et al., 2018), which can even utilize polymeric substrates like xylan and cellulose (Gardner, 2016), or *Pseudomonas stutzeri*, which was used to produce rhamnolipids under anaerobic conditions (Zhao et al., 2015; Table 1).

## Tailor-Made Rhamnolipids—Future Perspectives

The terms “tailor-made” or “designer rhamnolipids” initially were introduced to claim the possibility to freely choose the numbers of L-rhamnose sugar and 3-hydroxyfatty acid residues in the desired rhamnolipids. Mono-rhamnolipids are exclusively produced by expression of *rhlAB*, but expression of *rhlABC* typically results in a mixture of mono- and di-rhamnolipids. Several strategies from enzyme design and pathway modifications to congener enrichment by purification can be used to increase the purity of di-rhmnolipids (Manso Pajarron et al., 1993; Tiso et al., 2017; Wittgens and Rosenau, 2018). Moreover, the identification of the α/β-hydrolase(s), which synthesize mRmL and dRmL using typical mRdL and dRdL as precursors, would open up the possibility to specifically produce these rare rhamnolipid species. This would enlarge the portfolio of different available rhamnolipid species with specific physico-chemical properties for several applications. The so far only other known rhamnolipid modifying enzyme is the naringinase from *Aspergillus niger*, which removes a single L-rhamnose residue from di- and mono-rhamnolipids and finally generates HAAs (Trummler et al., 2003).

Another purpose for tailor-made rhamnolipids is to define the fatty acid chain lengths for each of the four species and possibly their degree of saturation customized for any type of specific applications. So far most of the publications used *rhl* genes from *P. aeruginosa* for the heterologous production of rhamnolipids in different hosts and only a few reports about the successful heterologous expression of *rhl* genes from *Burkholderia* (Wittgens et al., 2018; Dulcey et al., 2019). However, it has been described for *Pseudomonas desmolyticum* to produce rhamnolipids with predominant C_6_-C_8_ fatty acids (Jadhav et al., 2011), while other bacteria of the genus *Thermus* produce much longer rhamnolipids with up to 24 carbon atoms and a predominant C_16_-C_16_ congener (Řezanka et al., 2011), but none of them were produced heterologously so far. Germer et al. (2020) identified additional and partly unknown *rhlA* variants from different organisms by homology search. The heterologous expression of five of these *rhlA* variants in *E. coli* resulted in the production of two novel HAA congener compositions different from the typical composition of *Pseudomonas* and *Burkholeria* with its predominant C_10_-C_10_ and C_14_-C_14_ congeners. RhlA from *P. fluorescens* LMG 05825 produces HAA with 49% of a predominant C_10_-C_14_ congener and a slightly less C_10_-C_12_ congener of about 38%, while HAAs produced by RhlA from *Dickeya dadantii* Dd586_2334 contains a C_10_-C_14_ congener with 73%. These HAA mixtures could easily processed subsequently into mono- and di-rhamnolipids by additional expressions of appropriate *rhlB* and *rhlC*. Especially hybrid operons combining *rhl* genes from different organisms could give novel insights into the responsibility for defining rhamnolipid chain length. Such an approach using hybrid *rhlAB* operons from *P. aeruginosa* and *B. glumae* verified, that RhlA seems to mainly determine the fatty acid lengths used for the biosynthesis first of HAAs, but subsequent also for rhamnolipids (Wittgens et al., 2018). Similar experiments using the two originating species as hosts for such hybrid operons further indicated an influence of the host organism and its provided 3-hydroxyfatty acids (Dulcey et al., 2019).

A further possibility to achieve novel chain lengths in rhamnolipids is to manipulate the involved enzymes by single amino acid or whole domain exchanges to alter the accepted length of 3-hydroxyfatty acids as it was recently done. Thereby, Dulcey et al. (2019) created a chimeric RhlA variant using different original enzyme domains for RhlA originating form *P. aeruginosa* and *B. glumae*. Combined in an operon with *rhlB* from *P. aeruginosa*, the newly designed enzyme produced rhamnolipids with a predominant C_12_-C_12_ congener when heterologous expressed in *B. glumae*, which is the average between the typical short-chain rhamnolipids from *Pseudomnas* and the long-chain rhamnolipids from *Burkholderia*. In contrast, if the same operon was expressed in *P. aeruginosa* as host, the amount of C_12_-C_12_ congener was only increased in a mixture with a still predominant C_10_-C_10_ congener. It was further demonstrated, that mutagenized RhlA reached activities more than twice as much as the wild type RhlA (Dulcey et al., 2019) and amino acid exchanges of RhlB resulted in a shifted pattern of the rhamnolipid composition from C_10_-C_10_ to C_10_-C_8_ congeners indicating a further specificity of RhlB to specific HAAs containing certain fatty acid length (Han et al., 2014). Further enzyme modifications could result in a much more specific congener enriched composition through improved specificity of responsible enzymes to specific fatty acid chain lengths.

These strategies will help to develop rhamnolipids with their structural diversity as a platform molecule, which harbors an enormous potential to adopt tailor-made properties to meet a huge variety of demands of surfactants for various applications.

## Discussion

A specific heterologous host provides a given genetic and metabolic background to investigate the influence of different *rhl* genes by introducing specific or synthetic biosynthesis pathways for the production of various rhamnolipid species and congeners. Many different recombinant host organisms were established in the past belonging typically and with only a few exceptions to beta- or gamma-proteobacteria like the wild type producers.

A fundamental requirement to any rhamnolipid producing organism is its resistance against high rhamnolipid concentrations. This is most probably the reason, why none of the rhamnolipid producers is a Gram-positive organism, because rhamnolipids possess antimicrobial properties especially against Gram-positives (Abalos et al., 2001; Haba et al., 2003). Obviously, their single cytoplasmic membrane surrounded by the peptidoglycan layer does not represent an effective barrier against the influence of rhamnolipids (Sotirova et al., 2008, 2012). Recombinant *P. putida* appeared to be a good choice for heterologous rhamnolipid production, since with this organism the highest rhamnolipid titers of about 15 g/L could be achieved (Beuker et al., 2016a), while without using a profound bioprocess strategy titers of only a few hundred mg/L were reported for *E. coli* (Cabrera-Valladares et al., 2006; Wang et al., 2007; Du et al., 2017). However, none of the heterologous rhamnolipid producers so far reached the at least 40 g/L, which can reproducible be achieved using *P. aeruginosa* (Müller et al., 2011).

The most critical demand on heterologous rhamolipid producer so far is to provide sufficient amounts of educts. Gram-negative bacteria typically synthesize dTDP-L-rhamnose as they are part of the lipopolysaccharides in the other membrane of these bacteria (Rahim et al., 2000; Poon et al., 2008). However, the amount of this educt appears to limit heterologous rhamnolipid production especially in *E. coli* (Cabrera-Valladares et al., 2006) and probably other host organisms, but through suitable strategies like the coexpression of the *rmlBDAC* operon the amount of this educt can be increased.

The introduction of foreign and especially synthetic promoters enabled not only the circumvention of native quorum-sensing dependent regulation of rhamnolipid biosynthesis, but also the possibility to optimize and fine-tune the expression of responsible genes. This strategy will also allow to uncouple additional genes from their native regulation, e.g., responsible for the educt synthesis, which are usually strongly regulated in the wild types (Aguirre-Ramírez et al., 2012), to further improve the rhamnolipid biosynthesis. Metabolic engineering was also successfully used to lower the intrinsic metabolic burden by deleting high energy or resource-demanding side activities, e.g., the biosynthesis of polyhydroxyalkanoate (PHA) and the formation of flagella, which strongly increased the amounts of rhamnolipids (Wittgens et al., 2011; Tiso et al., 2016, 2020). This strain engineering in conjunction with strategies for bioprocess development (feeding strategies, media compositions, downstream processing, etc.) will contribute to lower the production cost for rhamnolipids and make them a more economical alternative in the future.

Moreover, the use of different rhamnolipid producer strains or more preferable the heterologous expression of the responsible genes already allows the production of several rhamnolipid species and congeners. Furthermore, real tailor-made rhamnolipids will probably become available soon through the identification and expression of novel rhamnolipid processing enzymes or enzyme modifications leading to a considerable enlargement of the portfolio of diverse rhamnolipids with the possibility to freely choose the chemical entities they consist of. This can assign a decisive role to rhamnolipids not only as the first class of true “designer” biosurfactant *per se*, but also as the first group of biosurfactants that can be produced exclusively in biotechnological processes at the same time.

## Author Contributions

AW and FR created figures and drafted the manuscript. All authors contributed to the article and approved the submitted version.

## Conflict of Interest

The authors declare that the research was conducted in the absence of any commercial or financial relationships that could be construed as a potential conflict of interest.
